# A Joining Process between Beryllium and Reduced-Activation Ferritic–Martensitic Steel by Plasma Sintering

**DOI:** 10.3390/ma14216348

**Published:** 2021-10-23

**Authors:** Jae-Hwan Kim, Taehyun Hwang, Masaru Nakamichi

**Affiliations:** Fusion Energy Directorate, Rokkasho Fusion Institutes, Breeding Functional Materials Development Group, National Institutes for Quantum Science and Technology, Aomori 039-3212, Japan; kim.jaehwan@qst.go.jp (J.-H.K.); nakamichi.masaru@qst.go.jp (M.N.)

**Keywords:** beryllium, F82H, plasma sintering, intermetallic compound, bonding strength

## Abstract

To investigate the growth kinetics of the reaction layer and mechanical strength of joined materials, we joined beryllium and reduced-activation ferritic–martensitic steel (F82H) by plasma sintering under various conditions and characterized the joined region. Scanning electron microscopy revealed that the thickness of the reaction layer increased with an increase in the joining time and temperature. Line analyses and elemental mapping using an electron microprobe analyser showed that the reaction layer consists of Be–Fe intermetallic compounds, including Be_12_Fe, Be_5_Fe, and Be_2_Fe, with small amounts of chromium and tungsten. Owing to the time and temperature dependence of the reaction-layer thickness, the layer growth of Be–Fe intermetallic compounds obeys the parabolic law, and the activation energy for the reaction-layer growth was 116.2 kJ/mol. The bonding strengths of the joined materials varied inversely with the thickness of the reaction layer.

## 1. Introduction

Great attention has been paid to technologies for joining materials, including metals, ceramics, polymers, and composites. Joining processes are promising techniques to improve specific properties, such as structural strengths and functional properties; the joined materials show more excellent properties than the individual materials [1,2]. Several techniques have been proposed for joining metals, including metallic brazing [3], welding [4], plasma spraying [5], and coating [6].

For fusion applications, beryllium (Be) is used as an amour for the first wall in an International Thermonuclear Experimental Reactor (ITER) owing to the advantages of low-atomic-number (Z) materials, including reduced impurity content, low loss of plasma radiation, and absence of chemical sputtering. However, Be forms intermetallic compounds with iron (Fe) [7], which may reduce the strength of the joint area since intermetallic compounds are considerably brittle. 

Efforts have been made to simplify the blanket design [8] by replacing the pebbles with block-shaped neutron-multiplying materials. This can also reduce the fraction of structural steel, which may contribute to plasma disruption or electromagnetic issues. When neutron-multiplying materials are used as block shapes by partial replacement of structural materials, the obtained material has good bonding strength to structural materials, such as reduced-activation ferritic–martensitic steel. Supposing the operating temperature is not high, Be can be considered the first wall material and as a block-shaped material in the blanket region of the fusion reactor to increase the neutron-multiplying efficiency.

Hot-isostatic-press (HIP) joining Be and F82H (Japanese reduced-activation ferritic–martensitic steel) has been conducted, and the thickness and bonding strengths of the reaction layers, such as thin Cr [9] and Ti films [10] have been investigated. The authors have, for the first time globally, fabricated Be [11,12] and beryllium intermetallic compounds [13,14,15] by plasma sintering, which is an advantageous process including surface activation for cleaning, and rapid heating and cooling speeds.

In this study, to clarify the growth kinetics of reaction layers and investigate the interfacial properties between joint areas, Be and F82H were joined by plasma sintering at various temperatures, and the mechanical properties of the joined materials were tested.

## 2. Materials and Methods

The starting material was F82H (Fe–8Cr–2W–0.2V–0.04Ta–0.1C), which is a candidate structural material for a Japanese test blanket material and demonstration (DEMO) reactor. The F82H (BA12 heat, ID:BT2-1-4) was treated by normalizing at 1313 K for 40 min and then tempered at 1023 °C for 60 min after hot rolling of an ingot material, and consisted of a tempered martensite structure [16]. The material was machined to a thickness of 17 mm and diameter of 20 mm with cutting accuracy of ±1 μm by an electrical discharge machine (Robocut α-OiD, FANUC, Yamanashi, Japan). Be materials (S65, Materion, Mayfield Heights, OH, USA) of the same thickness and diameter were machined to a cylindrical rod with a diameter of 20 mm and thickness of 17 mm. The joining areas of both F82H and Be samples were polished by up to #4000 silicon carbide (SiC) paper. To perform joining of two cylindrical materials, a plasma sintering (KE-PAS III, KAKEN Co., Ltd., Ibaraki, Japan) process was applied. After the two materials were placed in a graphite punch-and-die system, the Be and F82H samples were joined by plasma sintering at different sintering temperatures (923, 1023, and 1123 K) and at a pressure of 50 MPa for 90 min. To investigate the interfacial difference in the cross-section of the Be–F82H joint and the variation in the composition of the reaction layer, electron probe microscopic analysis (JXA-8530F, JEOL, Tokyo, Japan) with point and line analyses was conducted. In parallel to the point and line analyses, elemental mapping was conducted on the reaction layer with Fe, Cr, W, and Be.

To evaluate the mechanical properties of joined materials, four-point bending tests were conducted by an universal tester (AGX-10kNVD, Shimadzu, Kyoto, Japan), based on JIS R1601 with a crosshead speed of 0.5 mm/min at room temperature using three samples per each condition of dimensions 3 mm × 3 mm × 35 mm, and polished using #1200 SiC paper. Furthermore, the fractural surface was observed by scanning electron microscopy (SEM)/EPMA (JXA-8530F, JEOL, Tokyo, Japan) to clarify the fracture behaviour of the joined samples.

## 3. Results and Discussion

**Figure 1:**
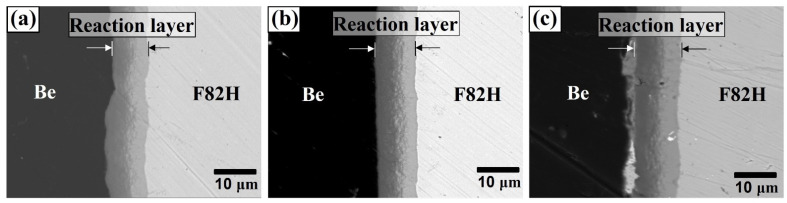
Cross-sectional SEM images of Be–F82H joints sintered at 1023 K for (**a**) 30 min, (**b**) 60 min, and (**c**) 90 min.

Figure 1 shows the cross-sectional SEM images of the Be–F82H interfacial region joined by plasma sintering at 1023 K for 30, 60, and 90 min. The reaction layers between Be and F82H joined by plasma sintering under all conditions were observed. The thickness of the reaction layers increased as time increased. For the material joined at 1023 K for 30, 60, and 90 min, the reaction layer thickness was 7.2, 9.3, and 12.8 μm, respectively.

**Figure 2:**
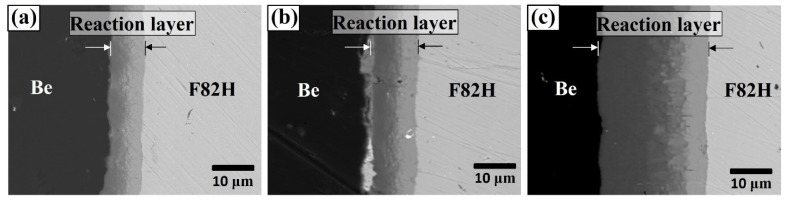
Cross-sectional SEM images of Be–F82H joints sintered at (**a**) 923 K, (**b**) 1023 K, and (**c**) 1123 K for 90 min.

To understand the temperature dependence of the reaction layer, the thicknesses of the reaction layers for the materials joined at 923, 1023, and 1123 K for 90 min were investigated, and the thickness was 6.8, 12.8, and 26.4 μm, respectively (Figure 2).

The thickness of a reaction layer between Be and F82H fabricated by the HIP process at 1023 K for 2 h was 6 μm [17], which slightly differs from the result herein. We speculate that plasma sintering facilitates the diffusion reaction more effectively than HIP since on–off direct current (DC) pulse promotes the sintering process, including effective discharge between particles of powder which leads to an electric field diffusion effect [18,19].

**Figure 3:**
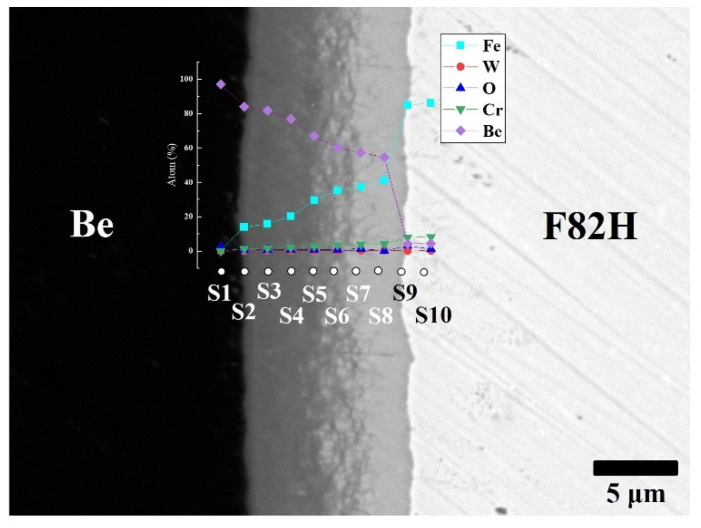
Cross-sectional SEM image of F82H–Be joints sintered at 1023 K for 60 min with point analyses from S1 to S10 with atomic % of Fe, W, O, Cr and Be.

To clarify the chemical composition of the reaction layers, SEM observation with backscattered electrons and point analyses were performed on the layers (Figure 3). The reaction layer consists mainly of Be and Fe elements and small amounts of Cr and W. The concentration gradient of Fe and Be was determined. This concentration gradient is attributed to the formation of different intermetallic compounds, including Be_12_Fe, Be_5_Fe, and Be_2_Fe (Table 1). The Be-enriched compounds detected at the measurement points went into the Be matrix.

**Figure 4:**
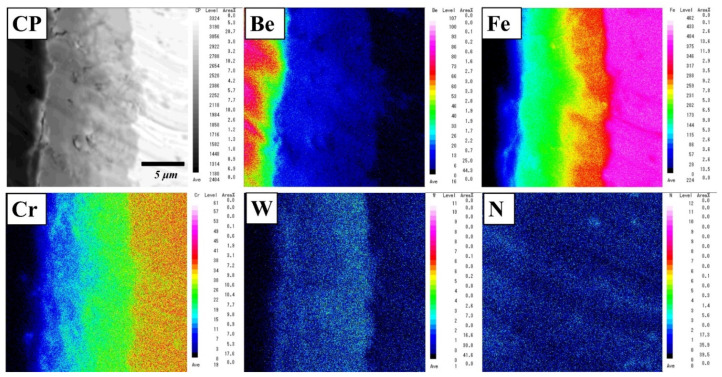
Elemental mapping of a reaction layer in a Be–F82H joint sintered at 1023 K for 90 min.

The analytical result is consistent with the elemental mapping of Be, Fe, Cr, W, and N elements using an electron microprobe analyser (EPMA) (Figure 4). The concentration gradient was dependent on the thickness of the layer. In the reaction layer, two or three layers with different compositions were identified. Be_12_Fe, Be_5_Fe, and Be_2_Fe with small amounts of Cr and W were detected (Table 1). Additionally, concentration gradients of Cr and W diffused from F82H were observed on the reaction layer. No remarkable depletion area was observed near the reaction layer.

**Figure 5:**
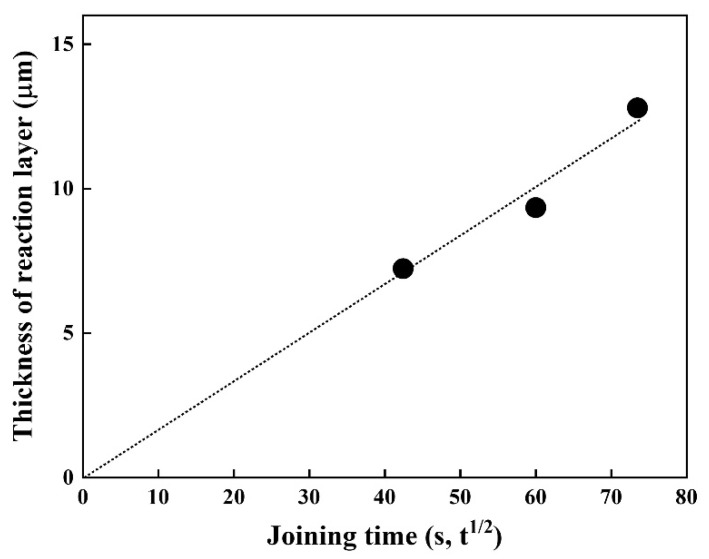
Thickness of the reaction layer as a function of joining time at 1023 K.

Since diffusion generally depends on time and temperature, it is vital to understand the joining time and temperature effect on the thickness of the reaction layer. Thus, the time dependence of the thickness of the reaction layer at 1023 K was investigated (Figure 5). The thickness of the reaction layer formed by reaction diffusion was proportional to the square root of the diffusion time (s), which is in good agreement with the results of previous studies [20,21]. The fitting result shows good linearity, implying the layer growth obeys the parabolic law, indicative of a diffusion-controlled growth, expressed as l = k·t^1/2^.

**Figure 6:**
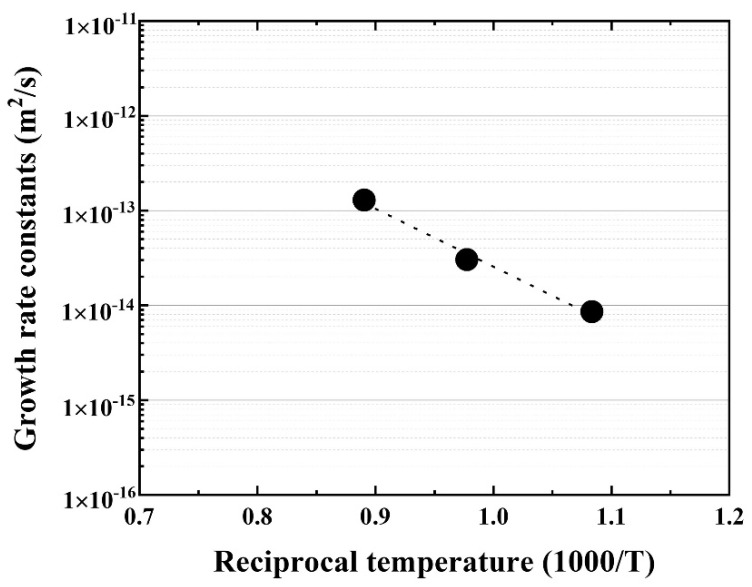
Arrhenius plot of the reaction layer as a function of reciprocal temperature.

Further, the temperature dependence of the reaction layer was evaluated using the materials joined at 923, 1023, 1123 K for 90 min, and the growth rate constant (m^2^·s) was calculated. The Arrhenius plot of the growth rate constants for the reaction layer is shown in Figure 6. 

**Figure 7:**
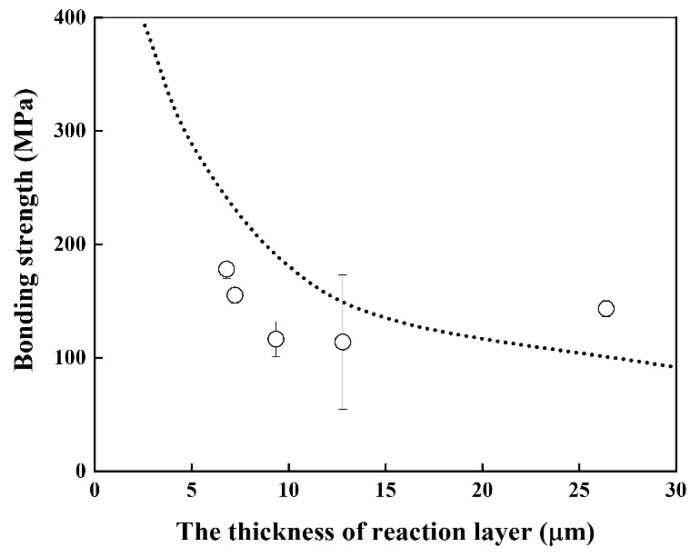
Bonding strength of joints as a function of the reaction layer thickness (Dot line indicates the result in [17]).

Good linearity was obtained, and the activation energy for the growth rate of the reaction layer was 116.2 kJ/mol, which is similar to that of Cu–Al intermetallic compound [22] and lower than that of β–Ti in Ti alloys [20]. The bonding strength of the Be/F82H joint was evaluated (Figure 7). It indicates that the bonding strength is inversely proportional to the thickness of the joint between Be and F82H. This inverse trend is because the increased thickness of the reaction layer comprising Be_12_Fe, Be_5_Fe, and Be_2_Fe decreased the bonding strength, which is in good agreement with the results in [17]. It is noted that all specimens were fractured at the joined area, in other words, it does not depend on the structure variation of the substrates, F82H and Be, even though those specimens were heat-treated at a temperature higher than the tempering temperature at 1023 K. 

**Figure 8:**
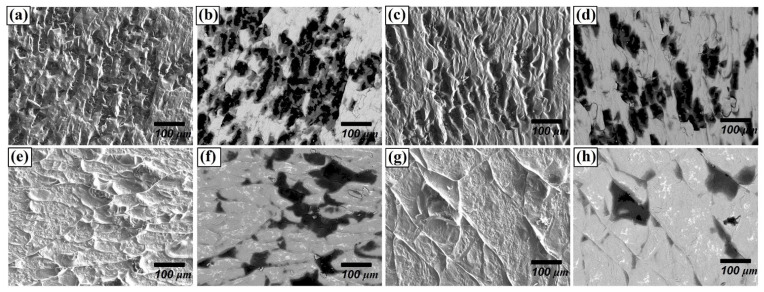
Fracture surface of F82H–Be joint at 1023 K for (**a**,**b**) 30 min, (**c**,**d**) 60 min, (**e**,**f**) 90 m, and (**g**,**h**) 1123 K for 90 min, (**a**,**c**,**e**,**g**: secondary electron images, **b**,**d**,**f**,**h**: backscattered electron images).

Regarding the fracture surface, for the materials joined for 30 min, many small-size grains were delaminated (Figure 8b) as seen with black colour. However, as the joining time and temperature increased, delaminations along the grain boundaries and the cleavage facets were observed. (Figure 8e,g), whereas the fraction of grain delamination decreased. This difference in delamination behaviour can be attributed to the variation in the bonding strength. It can be concluded that the increased thickness of the reaction layer comprising Be_12_Fe, Be_5_Fe, and Be_2_Fe decreased the bonding strength, since the intermetallic compounds are much more brittle than substrates.

## 4. Conclusions

Be and F82H were joined under various conditions to investigate the growth kinetics of the reaction layer and the mechanical strength of joined materials. As the joining time and temperature increased, the thickness of the reaction layer increased. Line analyses and elemental mapping by EPMA revealed that the reaction layer consists of Be–Fe intermetallic compounds, including Be_12_Fe, Be_5_Fe, and Be_2_Fe, with small amounts of Cr and W diffused from the F82H matrix.

Owing to the time dependence of the thickness of the reaction layer, the layer growth of Be–Fe intermetallic compounds predominantly obeys the parabolic law. Furthermore, by evaluating the temperature dependence of the thickness of the reaction layer, we found good linearity, and the activation energy for the growth of the reaction layer was 116.2 kJ/mol.

Finally, the bonding strength of the joined materials was inversely proportional to the thickness of the reaction layer. The difference in the fracture behaviour induced by either the delamination of grains or delamination along grain boundaries is attributed to the difference in the bonding strength.

## Figures and Tables

**Table 1:** Chemical composition (at.%) of each point and estimated compounds.

Measuring Point	Composition (at.%)					Estimated Compounds
	Be	Fe	Cr	W	O	
S1	97.16	0.44	0.02	0.00	2.38	Be
S2	84.03	14.02	1.23	0.10	0.62	Be_12_Fe
S3	81.91	15.80	1.50	0.12	0.68	Be_12_Fe
S4	76.88	20.25	1.89	0.17	0.80	Be_12_Fe, Be_5_Fe
S5	66.78	29.53	2.64	0.23	0.83	Be_5_Fe, Be_2_Fe
S6	60.21	35.25	3.40	0.32	0.82	Be_5_Fe, Be_2_Fe
S7	57.29	37.34	3.65	0.35	1.37	Be_2_Fe
S8	54.53	41.03	4.01	0.34	0.08	Be_2_Fe
S9	3.13	86.14	7.89	1.88	0.96	Be_2_Fe, F82H
S10	0.39	89.16	8.19	1.68	0.57	F82H

## Data Availability

Not applicable.
